# Prevalence of Microalbuminuria and Cardiovascular Risk Factors in Patients With Diabetes Mellitus Type-II in Al-Khobar, Kingdom of Saudi Arabia

**DOI:** 10.7759/cureus.29808

**Published:** 2022-10-01

**Authors:** Noor-Ahmed Jatoi, Abir H Said, Mawaddah S Al-Ghamdi, Marwah F Al-Abdulmhsin, Raghad A Bin-Jaban, Jumana A Al-Tayeb, Shadan A Aljarri, Ibrahim Saeed

**Affiliations:** 1 Internal Medicine, Imam Abdulrahman Bin Faisal University, King Fahd University Hospital, Al Khobar, SAU; 2 Internal Medicine, Imam Abdulrahman Bin Faisal University, King Fahd University Hospital, Al-Khobar, SAU; 3 Internal Medicine, College of Medicine, Imam Abdulrahman Bin Faisal University, Dammam, SAU; 4 Internal Medicine, Imam Abdulrahman Bin Faisal University, King Fahd University Hospital, Dammam, SAU

**Keywords:** type 2 diabetic mellitus (t2dm), cardiovascular risk factors (cvrf), glycated hemoglobin (hba1c), prevalence of cv risk factors, microalbuminuria

## Abstract

Background

Type 2 diabetes mellitus (T2DM) is a common disorder worldwide. Impaired control of glucose levels predisposes to renal dysfunction, detected by a diagnosis of microalbuminuria. Several other risk factors have been identified in the development of microalbuminuria, such as hypertension, smoking, dyslipidemia, and obesity.

Objective

Assessment of microalbuminuria and cardiovascular risk factors in type-II diabetic patients who attended the outpatient clinic for the internal medicine department at King Fahd University Hospital, Al-Khobar.

Methods

A retrospective cross-sectional and an observational study included data from 2014 to 2022 collected from medical records. Patients with diabetes type-II and aged ≥18 years were included. The following were reviewed (age, sex, height, weight, body mass index, waist, hip, waist-hip ratio, systolic and diastolic blood pressure, smoking, sedentary lifestyle, diagnosis of dyslipidemia/hypertension, diabetes duration in years) and laboratory results (fasting blood glucose, HbA1C%, estimated glomerular filtration rate, serum creatinine, serum cholesterol, low-density lipoprotein, high-density lipoprotein, and triglycerides). Microalbuminuria was measured by the urine albumin to creatinine ratio and was diagnosed if levels were 30-300 mg/g.

Results

Among 301 studied patients, the prevalence of microalbuminuria was found at 36.8%. The mean age was 57.8 ± 12.6 years, and females were 45%. The mean ± SD fasting blood glucose was 165.9 ± 71.9 mg/dL, while HbA1C% was 8.8 ± 5.6. Microalbuminuria was significantly associated with age, diabetes duration, systolic blood pressure, HbA1C%, fasting blood glucose, and triglyceride levels (p≤0.05).

Conclusion

Microalbuminuria in T2DM patients was high in this study, which emphasizes the need for early detection of microalbuminuria. The study suggests the need for effective diabetes control and the prevention of associated cardiovascular risk factors.

## Introduction

Microalbuminuria is an early representative of renal damage or nephropathy in diabetic patients. Microalbuminuria is defined as the increase in urine albumin excretion (30-300 mg/day) or (20-200 mg/day). Urine albumin/creatinine ratio (urine ACR) is the preferred screening approach. Microalbuminuria is diagnosed when urine ACR levels range from 30 to 300 mg/g [1]. Diabetic nephropathy affects around one-third of patients with type 2 diabetes mellitus (T2DM) in Saudi Arabia [2]. It has the highest rate when compared to the global incidence of end-stage renal disease caused by diabetic nephropathy [3]. Hence, screening for microalbuminuria in diabetic patients can help in the early detection of diabetic nephropathy and the prevention of further complications.

Hypertension, smoking, hyperglycemia, dyslipidemia, and overweight or obesity are all cardiovascular risk factors that have been associated with the development of microalbuminuria [4]. Glycation of the glomerular basement membrane can occur as a consequence of chronic hyperglycemia and inadequate glycemic management. This, in turn, leads to the progression of diabetic nephropathy as a result of a high glomerular filtration rate and low filtration capacity [5]. Hypertension has been associated with a higher risk of cardiovascular disease. As a result, it causes proteinuria and reduced renal function. Albumin exertion in the urine has been linked to hydrostatic pressure alterations in the afferent glomerulus, increased leakage of the glomerular basal membrane, and poor tubular function in hypertensive patients [6].

Recent studies have concluded that diabetic nephropathy progression is associated with dyslipidemia. High levels of blood total cholesterol (TC), low-density lipoprotein (LDL), serum triglycerides (TG), and low levels of high-density lipoprotein (HDL) characterize diabetic dyslipidemia [7]. Since albuminuria is substantially related to high levels of total cholesterol, the presence of dyslipidemia can be used as an assessment tool for microalbuminuria among patients with T2DM [8]. Cigarette smoking has an independent association with increasing levels of urine albumin. When compared to non-smokers, patients with T2DM who were current smokers had an increased incidence of microalbuminuria per year [9].

Body mass index (BMI) and waist-to-hip ratio (WHR) have been significantly associated with microalbuminuria [10]. The specific pathogenesis regarding obesity-related glomerulopathy is hyperfiltration, which is mediated by excessive protein and salt intake, high blood insulin levels, and increased feedback of the tubuloglomerular component. Central obesity is associated with the secretion of active proteins and proinflammatory cytokines, which play a role in renal injury [11]. Although the previously mentioned metabolic irregularities contribute to renal impairment, there is little evidence regarding the relationship between WHR and microalbuminuria in patients with T2DM as has been established in one study [12]. Hence, studying the association between central obesity and microalbuminuria is one of the main goals of our research. The current study aims to estimate microalbuminuria prevalence and assess the related cardiovascular risk factors among patients with T2DM in Al-Khobar, Saudi Arabia.

## Materials and methods

This study is a retrospective, cross-sectional study conducted on patients with T2DM who attended the department of internal medicine at King Fahd University Hospital (KFUH) Al-Khobar, Eastern Province, Saudi Arabia from January 2014 to December 2021. Ethical approval from the Imam Abdulrahman Bin Faisal University Institutional Review Board was obtained.

The study comprised 301 patients with T2DM. Inclusion criteria: patients diagnosed with T2DM, aged ≥18 years, both males and females were included. Exclusion criteria: patients aged <18 years, previously diagnosed with diabetes type-I, congestive heart failure, chronic kidney disease, or nephrotic syndrome, any patient with a history of steroid use, exposure to radiocontrast agents, active urinary tract infection, fever or pregnancy at the time of urine ACR test was excluded from the study.

Demographic, anthropometric, clinical and laboratorical information for the included patients were reviewed and retrieved from the KFUH database, Quadra Med Computerized Patient Record (QCPR). Past medical history including the duration of diabetes and the diagnosis of hypertension and dyslipidemia were taken from outpatient clinic visit notes. Age, gender, height, weight, and body mass index (BMI), which was calculated and classified into normal (<25), overweight (25-29.9), obese (30-39.9), and morbidly obese (>40), the circumference of waist and hip, waist-hip ratio, smoking status, sedentary lifestyle (inactive) [13]. The readings included were systolic blood pressure, diastolic blood pressure, and pulse pressure. HbA1C% and fasting blood glucose (FBG) were used for the assessment of glucose control. HbA1C% was classified as controlled (<7%) and uncontrolled (≥7%), while fasting blood glucose readings were divided into normal (<130 mg/dL) and abnormal (≥ 130 mg/dL) [14]. Patients’ lipid profiles for dyslipidemia assessment included total cholesterol, LDL, HDL, and triglycerides. Other important laboratory data for renal profile were serum creatinine and estimated glomerular filtration rate (eGFR), which were calculated by the Modification of Diet in Renal Disease (MDRD) study equation (175 × standardized Scr −1.154 × age−0.203 × 1.212 (if black) × 0.742 (if female)) [15]. eGFR results were classified by the National Kidney Foundation into the following (normal/high ≥90, mildly decreased 60-80, mildly-moderately decreased 45-59, moderately-severely decreased 30-44) [16]. Microalbuminuria was detected according to hospital policy by using a random spot urine sample. The urine albumin-creatinine ratio was calculated by dividing albumin concentration in milligrams by creatinine concentration in grams. A value of ≥30-300 mg/g was considered positive microalbuminuria [17]. Statistical analysis was performed by using SPSS Statistics for Windows, version 26.0 (IBM SPSS Statistics for Windows, IBM Corp, Armonk, NY).

## Results

Table 1 shows the demographic features and associated variable characteristics of all 301 included patients. The mean age was 57.76 ± 12.64 years. Males (55.1%) were slightly more than females in number. The majority of the patients never smoked, while (25.6%) were smokers. Most of the patients (64.1%) had dyslipidemia. About 57% had hypertension, which is less than expected. Sedentary lifestyle was noticed among more than half of the patients. Most of the participants had high BMI readings. The mean waist-hip ratio was ≥0.9, which is in the high-risk category for metabolic complications. As for the T2DM duration, most of the patients (64.1%) had been diagnosed with T2DM for more than five years. While diastolic and pulse pressure were in the normal range with values of 78.91 ± 11.55 and 55.38 ± 18.21, respectively. The mean systolic blood pressure was high at 136.2 ± 19.43. For blood glucose control, the majority had uncontrolled diabetes (HbA1C of ≥7%) with a mean level of 8.82 ± 5.58. While fasting blood glucose was abnormal at 66.1%, with a mean level of 165.9 ± 71.94. The mean urine albumin, urine creatinine, and urine ACR were in accordance with microalbuminuria findings. While serum creatinine was within the normal range for most of the patients, about 43.5% had a mildly decreased (60-80) eGFR (mL/min/body surface area (BSA)) with a mean level of 81.11 ± 25.33. More than half of the patients (64.1%) had high cholesterol, LDL, TG levels, and low HDL levels. 

**Table 1:** Relationship between microalbuminuria and patients’ demographic features and associated variables characteristics. T2DM: type 2 diabetes mellitus. *Significant association.

Variable	Urine microalbuminuria level mean ± SD/(%)		p-value
	Normal	Microalbuminuria	
Age	55.7 ± 12.3	61.4 ± 8.3	0.002*
Triglycerides (mg/dL)	132.2 ± 64.8	165 ± 94.5	0.041*
HbA1C (%)	8.4 ± 1.8	8.9 ± 1.8	0.031*
Fasting blood glucose	160 ± 81.6	176.5 ± 71.9	0.015*
Normal (<130 mg/dL)	(76.8)	(23.2)	0.008*
Abnormal (≥130 mg/dL)	(55.2)	(44.8)	
T2DM duration in years			
<5	(61)	(39)	0.03*
5-10	(73.2)	(26.8)	
10-15	(81)	(19)	
>15	(49)	(51)	

Table 2 shows a prevalence of 36.8% for microalbuminuria. Patients with older age, T2DM duration of more than 15 years, elevated systolic BP, high HbA1C (≥7%), abnormal FBG (≥130 mg/dL), and high TG levels were found to have a significantly higher percentage of microalbuminuria (p ≤ 0.05). Whereas the relationship between microalbuminuria and other patients’ socio-demographic or disease-related characteristics was found to be non-significant (p ≥ 0.05). 

**Table 2:** Diabetics’ demographic features and associated variable characteristics (n = 301). LDL: low-density lipoprotein, HDL: high-density lipoprotein, FBG: fasting blood glucose, ACR: albumin/creatinine ratio, eGFR: estimated glomerular filtration rate, BSA: body surface area, BMI: body mass index, T2DM: type 2 diabetes mellitus.

Variable		Mean ± SD/(%)
Age		58 ± 13
Male: female		(55:45)
Smoker, never smoked		(25.6), (74.4)
Dyslipidemia		(64.1)
Total cholesterol (mg/dL)		165.3 ± 45.7
LDL (mg/dL)		101 ± 39.5
HDL (mg/dL)		45.1 ± 13.7
Triglycerides (mg/dL)		143 ± 86.6
Hypertension		(57.5)
Systolic BP (mmHg)		136.2 ± 19.4
Diastolic BP (mmHg)		78.9 ± 11.6
Pulse pressure (mmHg)		55.4 ± 18.2
Sedentary lifestyle (inactive)		(52.8)
BMI		32.2 ± 7.7
Height (m)		1.6 ± 0.1
Weight (kg)		85.1 ± 19.4
Waist-hip ratio (WHR)		1 ± 0.1
Waist (cm)		110.1 ± 14.9
Hip (cm)		110.2 ± 12.6
HbA1C (%)		8.8 ± 5.6
Controlled <7%		(23.9)
Uncontrolled ≥7%		(76.1)
Fasting blood glucose		165.9 ± 71.9
Normal FBG (<130 mg/dL)		(33.9)
Abnormal FBG (≥130 mg/dL)		(66.1)
Urine albumin (mg/dL)		11.1 ± 25.2
Urine creatinine (mg/dL)		128.5 ± 94.2
Urine ACR (mg/g)		55.1 ± 216.9
Serum creatinine (mg/dL)		1.1 ± 2.7
eGFR (mL/min/BSA)		81 ± 25.3
Normal or high (≥90)		(38.2)
Mildly decreased (60-80)		(43.5)
Mildly to moderately decreased (45-59)		(10.6)
Moderately to severely decreased (30-44)		(7.6)
T2DM duration in years		
<5	(35.9)	
5-10	(23.6)	
10-15	(11.3)	
>15	(29.2)	

Table 3 represents the correlation of urine ACR levels and microalbuminuria with all the numerical variables. A correlation with age, T2DM duration, pulse pressure, FBG, HbA1C, and TG levels was found to be significantly positive (p ≤ 0.05).

**Table 3:** Spearman's rank correlation for urine albumin/creatinine ratio and microalbuminuria. T2DM: type 2 diabetes mellitus. *Pearson’s correlation.

Variable	Urine albumin/creatinine ratio		Microalbuminuria	
	r^2^	p-value	r^2^	p-value
Age (years)	0.19	0.018	−0.05	0.698
T2DM duration (years)	0.18	0.043	−0.01	0.514
Systolic BP (mmHg)	0.24*	0.003	0.24	0.073
Pulse pressure (mmHg)	0.32*	<0.001	0.026	0.047
Fasting blood glucose (mg/dL)	0.27	0.001	0.3	0.02
HbA1C (%)	0.24	0.002	0.33	0.012
Triglecirides (mg/dL)	0.2	0.011	0.38	0.003

Table 4 represents the correlation of FBG and HbA1C levels with all the numerical variables. A correlation with T2DM duration, hip-waist-ratio, urine creatinine, urine albumin, total cholesterol, and TG was found to be significantly positive (p ≤ 0.05).

**Table 4:** Spearman's rank correlation for fasting blood glucose and HbA1C. T2DM: type 2 diabetes mellitus.

Variable	Fasting blood glucose (mg/dL)		HbA1C (%)	
	r^2^	p-value	r^2^	p-value
T2DM duration (years)	0.11	0.123	0.14	0.043
Hip-waist-ratio	0.16	0.045	−0.15	0.057
Urine creatinine (mg/dL)	−0.19	0.015	−0.06	0.44
Urine albumin (mg/dL)	0.16	0.047	0.24	0.003
Total cholesterol (mg/dL)	0.08	0.151	0.15	0.01
Triglecirides (mg/dL)	0.15	0.01	0.24	<0.001

## Discussion

Detection of microalbuminuria is one of the crucial steps in managing diabetic patients as it is considered an early sign of renal impairment and subsequent diabetic nephropathy [1]. In this study, 301 diabetic patients were investigated for microalbuminuria and its associated risk factors. The screening for microalbuminuria was conducted using a random sample of urine to test for urine ACR, which is the recommended screening method for microalbuminuria [1]. This study’s microalbuminuria prevalence was 36.8%. Previous studies showed a similar result, which was reported in multiple cross-sectional studies in Saudi Arabia ranging from 33.2% to 41.3%, 31.8% to 34.2% in Egypt, and 39% in the DEMAND study globally [17-22].

Despite the similar microalbuminuria prevalence in the current study, there is a possibility of variation in prevalence based on a number of factors related to differences in the population characteristics, microalbuminuria definition, measurement methods, and collection of urine [17]. Gender-specific association with microalbuminuria has been reported in several studies. However, there were a few studies that reported no statistical significance between gender and microalbuminuria [19,22-24]. Our study shows a similar result, in which there was no statistical significance between gender and microalbuminuria. A male predominance in the prevalence of microalbuminuria was reported [8,25,26]. While many other studies reported that the female gender was associated with microalbuminuria [17,18,21,27].

Multiple cross-sectional studies found no association between age and microalbuminuria [17,19,23-26,28]. On the contrary, a few studies reported a statistical significance between age and microalbuminuria [8,21]. In the present study, age was observed to be significantly related to microalbuminuria. The present study showed a significant association of longer diabetes duration with the presence of microalbuminuria, which was similar to a previous study [18]. This could be justified by how the perpetuation of hyperglycemia would result in the build-up of glycosylation end products and the presence of protein in urine [5].

Chronic hyperglycemia leads to a decline in renal function by glycation of glomerular basement membranes and stiffening of efferent arterioles. This will adversely increase the glomerular filtration rate, regress filtration capacity and give rise to diabetic nephropathy [5]. HbA1C ≥7% is an indicator of poor glycemic control. The mean value of HbA1C% for the microalbuminuria group was (8.95 ± 1.79) compared to (8.4 ± 1.84) in patients with normal urine ACR. This study showed a significant association of uncontrolled HbA1C with microalbuminuria similar to a study done in Taif, Saudi Arabia [23]. The mean value of abnormal FBG ≥130 among patients with microalbuminuria was (176.5 ± 71.87) while for non-microalbuminuria patients it was (159.99 ± 81.63). A significant relationship was noticed among the microalbuminuria group as it was found in other studies [19,20]. Poor glycemic control is a widely known cause of diabetic nephropathy. Our findings were consistent with a previous study [25].

Hypertension is a recognized risk factor for renal impairment [6]. This study has shown a nonsignificant relationship between hypertension diagnosis and microalbuminuria. A similar finding was noted in Alzaid et al., where hypertension had no significance on microalbuminuria [19]. While the contrary was found in several studies [21,23,25,29]. On the other hand, high systolic blood pressure was found to be significantly associated with the presence of microalbuminuria in this study, as seen in a similar study [29]. Increased systolic blood pressure negatively impacts renal function by affecting the systemic arterioles and constant insult to the glomerular filtration membrane resulting in microalbuminuria [30].

Dyslipidemia is a well-studied risk factor for diabetic nephropathy [7]. Previous studies demonstrated a statistical significance regarding the high levels of LDL with the occurrence of microalbuminuria [17,27]. Showail et al. reported a statistical significance between the high levels of total cholesterol, triglycerides, and microalbuminuria [8]. Our study shows a similar result in which high levels of triglycerides were statistically significant with microalbuminuria. However, many studies showed non-statistical significance between lipid profile parameters and microalbuminuria [17,19,23,24,28].

Obesity-related glomerulopathy has been associated with hyperfiltration, which is mediated by excessive protein and salt intake, high blood insulin levels, and increased feedback on the tubuloglomerular component [11]. A few earlier studies demonstrated a statistical significance between BMI and microalbuminuria [17,21,28,26]. Many studies, on the contrary, have not reported statistical significance. This finding was seen in the DEMAND study globally, which was possibly supported by the fact that the Asian population had the lowest BMI despite the highest rates of microalbuminuria [22]. Other studies done in the Middle East showed a similar result of non-significance [8,18,19,23-26]. In the present study, BMI and microalbuminuria did not have a statistically significant relationship.

Central obesity, measured by waist-to-hip ratio, is associated with the secretion of active proteins and proinflammatory cytokines that have a role in renal injury [11]. Based on the literature review, only one study showed a statistical significance between high WHR and microalbuminuria in females with T2DM [12]. Most of the participants in the study were in the higher risk group for WHR. Our study failed to show an association of WHR with microalbuminuria.

Tobacco smoking is one of the independent risk factors for increasing levels of urine albumin [9]. Our study has shown a non-significant relationship with microalbuminuria as seen in other studies [22,24]. On the contrary, many studies reported tobacco use as a risk factor and had linked smoking to the development of microalbuminuria [21,22,25]. Our controversial findings could be attributed to the small sample size and low prevalence of female smokers.

Despite the good preparation of this study, there were a few limitations. The study participants were from a single hospital in the Al-Khobar area. Due to time restrictions, the sample size was very small to represent the prevalent number of diabetic patients in society. Furthermore, the data collection was clinic-based, so the result was exclusively observed for patients who were following up on a regular basis.

## Conclusions

Microalbuminuria is one of the important tests for early renal damage in T2DM patients. A prolonged duration of diabetes, an increase in age, poor diabetes control, an increased level of triglycerides, and high systolic blood pressure were associated with microalbuminuria. Thus, annual screening for microalbuminuria, maintaining good glycemic control, and managing cardiovascular risk factors can help in reducing microalbuminuria and the progression into diabetic nephropathy. This study encourages regular and early screening of microalbuminuria and the prevention of irreversible kidney damage that results from poor diabetic control. Moreover, the renal injury could be exacerbated by other cardiovascular risk factors such as dyslipidemia and hypertension. This study implements educational material to enhance patient awareness and understanding of their condition and how crucial diabetic control is for reducing complications.
